# Extracellular Vesicle Proteins and MicroRNAs as Biomarkers for Traumatic Brain Injury

**DOI:** 10.3389/fneur.2020.00663

**Published:** 2020-07-16

**Authors:** Vivian A. Guedes, Christina Devoto, Jacqueline Leete, Delia Sass, Jedidiah D. Acott, Sara Mithani, Jessica M. Gill

**Affiliations:** National Institute of Nursing Research, National Institutes of Health, Bethesda, MD, United States

**Keywords:** exosomes, concussion, neurodegeneration, neuroinflammation, extracellular vesicles

## Abstract

Traumatic brain injury (TBI) is a heterogeneous condition, associated with diverse etiologies, clinical presentations and degrees of severity, and may result in chronic neurobehavioral sequelae. The field of TBI biomarkers is rapidly evolving to address the many facets of TBI pathology and improve its clinical management. Recent years have witnessed a marked increase in the number of publications and interest in the role of extracellular vesicles (EVs), which include exosomes, cell signaling, immune responses, and as biomarkers in a number of pathologies. Exosomes have a well-defined lipid bilayer with surface markers that reflect the cell of origin and an aqueous core that contains a variety of biological material including proteins (e.g., cytokines and growth factors) and nucleic acids (e.g., microRNAs). The presence of proteins associated with neurodegenerative changes such as amyloid-β, α-synuclein and phosphorylated tau in exosomes suggests a role in the initiation and propagation of neurological diseases. However, mechanisms of cell communication involving exosomes in the brain and their role in TBI pathology are poorly understood. Exosomes are promising TBI biomarkers as they can cross the blood-brain barrier and can be isolated from peripheral fluids, including serum, saliva, sweat, and urine. Exosomal content is protected from enzymatic degradation by exosome membranes and reflects the internal environment of their cell of origin, offering insights into tissue-specific pathological processes. Challenges in the clinical use of exosomal cargo as biomarkers include difficulty in isolating pure exosomes, variable yields of the isolation processes, quantification of vesicles, and lack of specificity of exosomal markers. Moreover, there is no consensus regarding nomenclature and characteristics of EV subtypes. In this review, we discuss current technical limitations and challenges of using exosomes and other EVs as blood-based biomarkers, highlighting their potential as diagnostic and prognostic tools in TBI.

## Introduction

Traumatic brain injury (TBI) is a major cause of disability worldwide, affecting an estimated 10 million people annually, representing a growing burden to public health (1, 2). TBI may be caused by a bump, blow or jolt to the head or a penetrating head injury that causes structural damage or disrupts normal brain function (3). TBI severity can range from mild to severe and is determined based on clinical factors including presence and duration of loss of consciousness, post-traumatic amnesia, mental state alterations and neuroimaging findings (3). Mild TBI (mTBI) is the most frequent type, affecting all demographics (4). Clinical management of mTBI is challenging as diagnosis may be difficult and clinical presentation and recovery varies among individuals. Moreover, even in milder cases, TBI may trigger neurodegenerative changes and place survivors at risk of developing chronic neurological and behavioral symptoms (5, 6), and affecting quality of life and functioning of individuals within family and society (7).

TBI is increasingly seen as a chronic disorder that may affect long-term health (7). Factors underlying individual susceptibility to develop TBI-related neurodegenerative changes and persistent or late-in-life symptoms are still largely unknown (7, 8). Lifestyle, sex, genetic, and social factors, medical history, including previous head injuries, are all likely important determinants in TBI recovery (7, 9). Indeed, sustaining multiple TBIs has been linked to lasting or worsening neurobehavioral symptoms, placing populations such as service members and contact-sport athletes at a higher risk for worse outcomes following a TBI (10–12). The heterogeneous nature of TBI and limited understanding of underlying pathology represents a challenge to the development of effective therapeutic strategies. Thus, the clinical need for diagnostic and prognostic tools for TBI has prompted several studies aimed at identifying biomarkers to inform clinical interventions and identifying those most at risk for poor recovery and chronic sequelae (13–17). Candidate biomarkers measured in serum or plasma (i.e., blood-based biomarkers) and other bodily fluids have been explored by several research groups and offer safe and inexpensive methods to monitor brain injury (13–20). Most studies have focused on proteins derived from damaged neurons and astrocytes (13, 17, 21–23). Other candidate biomarkers include markers of inflammatory responses and vascular injury (14, 24, 25), as well as circulating microRNAs (miRNAs), which are small non-coding RNAs with major roles in the regulation of gene expression (26, 27). Studies have shown the potential of biomarkers to inform clinical decisions and predict short-term outcomes such as return-to-play in sport-related concussion (14, 17, 23, 28).

In recent years, exosomes have sparked interest in the scientific community for their emerging role in cell-to-cell communication involved in physiological and pathological processes throughout the body. Exosomes are part of the broader population of extracellular vesicles (EVs), that also includes microvesicles and apoptotic bodies (29). However, no consensus has been reached among experts regarding characteristics of EV subtypes as this is a fast evolving and relatively new research field, and specific markers are still being defined (30–32). The terms exosome and EV are commonly used interchangeably in the literature to refer to vesicles formed by a lipid bilayer that contains cargo including proteins (e.g., cytokines and growth factors), nucleic acids and lipids (30, 32). Here, we follow the International Society for Extracellular Vesicles (MISEV) recommendations and adopt the term EV as the generic term for lipid bilayer-delimited particles released from cells (31, 32). Thus, the term EV will be used in this review to describe study findings and general concepts, applying the term exosome only when necessary to describe the specific subtype of EV.

Candidate biomarkers of TBI, including proteins and miRNAs previously identified in serum or plasma samples, have also been found in EVs isolated from peripheral blood (33, 34). Moreover, efforts have been made to identify the proteomic signature and RNA expression profiles in EVs derived from specific cell types (35–37). This review focuses on the evidence of EVs as a promising new family of biomarkers for TBI as well as challenges in the field. We address the emerging insights into roles played by EVs in the central nervous system (CNS), linking them to TBI-related neuropathology.

## Tbi-related Symptoms and Associated Disorders

TBI can result in highly variable symptoms among individuals that are typically related to physical, cognitive, and affective domains (38, 39). Headache is the most common physical symptom in individuals with mTBI (40, 41). Sleep disruption and fatigue are also frequently reported following TBI; incidents estimates indicate that post-TBI, between 21 and 73% of individuals experience fatigue, which can persist for years after initial injury (42). Some form of sleep disturbance is reported by 50% of individuals following TBI. Moreover, prevalence rates of sleep disorders in TBI patients are elevated compared to the general population, with two times the risk for periodic limb movements, three times the risk for insomnia and hypersomnia, and 12 times the risk for sleep apnea (43). Other common physical symptoms include dizziness (41, 44, 45), nausea (41), light/noise sensitivity (46), chronic pain (47), and in moderate-to-severe TBI, seizures (48).

Acute moderate to severe TBI is characterized by impaired consciousness and post-traumatic amnesia (PTA) (3, 49). In mTBI, loss of consciousness and PTA might not occur. Subacute and chronic cognitive symptoms are common, persisting in ~31–63% of individuals who sustained a TBI (50–52). The most prevalent chronic cognitive deficits are memory, executive functioning, attention, and processing speed, especially among those with a history of multiple mTBIs or moderate-to-severe TBI (51, 53, 54). Symptoms following TBI, even mTBI, can be long-lasting: more than half of patients who incurred a TBI reported experiencing three or more symptoms 1 year after injury (55). In mTBI, the collection of neuropsychological symptoms (i.e., a constellation of neurological, cognitive, and affective symptoms) is often referred to as post-concussion syndrome (PCS). However, this term is controversial and is not universally accepted because these symptoms are not specific to concussion patients and can be found in patients with moderate and severe TBI (55–58).

TBI is a risk factor for the development of several psychiatric disorders. Post-traumatic stress disorder (PTSD) and TBI are often comorbid, with this relationship mostly studied in military populations. In two studies of over 2,500 US military personnel, 44% of individuals who reported loss of consciousness during deployment also met criteria for PTSD (59); and combat-related mTBI increased the risk for PTSD more than 2-fold (60). Major depressive disorder (MDD) and generalized anxiety disorder (GAD) are frequently comorbid with TBI; 27 and 11% of individuals with TBI are also diagnosed with MDD or GAD, respectively (61, 62).

TBI, including mild cases, can lead to neurogenerative changes. Moderate to severe TBI has been linked to earlier onset of Alzheimer's disease (AD) and dementia (5, 6, 63–66). A recent meta-analysis concluded that previous head injury increases the risk factor for any dementia by 63% and AD by 51%, but only for males (67). Moreover, mTBIs have been recently associated with a 2-fold increased risk of developing dementia in Veterans (5). Multiple mTBIs are linked to elevated risk of developing progressive neurodegenerative disease associated with neurological and cognitive impairments (6). TBI, including mTBI, has also been associated with increased risk for Parkinson's disease (68, 69). Sustaining multiple mTBIs is linked to elevated risk of developing progressive neurodegenerative disease associated with neurological and cognitive impairments (6).

The link between TBI and a wide range of cognitive, psychiatric, and neurological symptoms and disorders is marked, but the biological processes underlying this association are still largely unknown. As discussed in the following sections, TBI results in a cascade of cellular and molecular events that lead to cell death, neurovascular injury and inflammation (3, 6). Several research groups have been able to isolate EVs from the peripheral blood of TBI patients and measure their content (33, 35, 37). Analyzing levels of specific proteins, miRNAs, and other signaling molecules in EVs at different timepoints after TBI, while examining relationships with specific symptoms, could lead to the development of novel therapeutic strategies in TBI, and biomarkers that predict risk of developing specific symptoms after a head injury. This approach may ultimately lead to clinical interventions for those most at risk, prior to the onset of symptoms and underlying pathological processes. Associations between EV biomarkers and symptom severity are described in the following sections (33, 35). Next, we discuss major mechanisms underlying TBI neuropathology.

## Neuropathology of TBI

The pathology of TBI is complex, heterogeneous, and comprised of both immediate and delayed elements. Morphologically, brain injury can be divided into focal and diffuse injury. Focal injury is due to a severe and direct impact on the brain, including cortical and subcortical contusions and lacerations as well as hemorrhage and hematoma (70, 71). Diffuse injury is caused by stretching and tearing of brain tissue and includes axonal and microvascular injury (70, 71). Diffuse axonal injury (DAI) is a form of diffuse injury caused by acceleration and deceleration forces that lead to the shearing of axons (70, 72). DAI is a key pathological process in mTBI, reflecting the vulnerability of white matter axons to rapid head acceleration/deceleration caused by a hit to the head. DAI is believed to break the axonal cytoskeleton, affecting axonal transportation, which leads to neurodegeneration (70, 72, 73).

**Figure 1:**
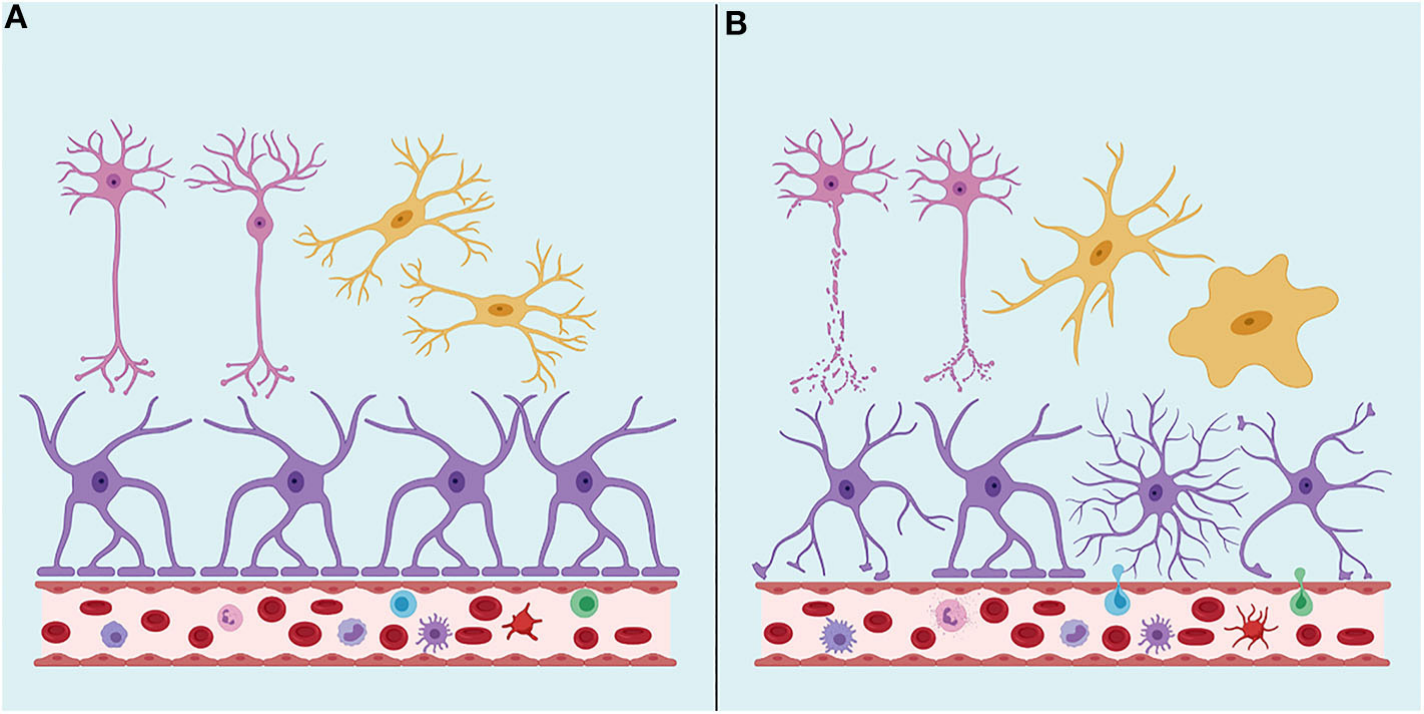
**(A)** Under physiological conditions, the Brain-Blood Barrier (BBB) creates a restrictive barrier between central nervous system and circulating blood contents. BBB is formed by astrocyte endfeet, pericytes and tight junctions among endothelial cells. **(B)** After a traumatic brain injury (TBI), BBB may become dysfunctional. TBI may also lead to axonal shearing, activation of microglia, astrocytes, and peripheral immune cells, often resulting in neuroinflammation, edema, neuronal hyperexcitability, and cell death. Neurons are represented in pink, astrocytes in purple and microglia in yellow. Created with Biorender.com.

TBI neuropathology consists of a primary injury, which ranges from mild to severe, that is a direct consequence of the traumatic insult and the effects of the mechanical forces on the brain tissue, directly damaging neurons, glial cells and vasculature in focal or diffuse patterns (71) (Figure 1). Secondary injury results from a cascade of molecular and cellular events triggered by the primary injury and includes responses such as edema, hypoxic-ischemic injury, vascular injury, hypometabolism, and neuroinflammation (70, 71).

Microglia are resident myeloid cells in the brain that clean debris and dying cells, among other housekeeping functions (74). Microglia mediate host defense against infectious pathogens, CNS tumors, and proteins such as amyloid β (Aβ) (75, 76). In response to TBI, microglia, as well as astrocytes, become active, changing morphology and initiating an inflammatory cascade by secreting cytokines, chemokines, and growth factors (76, 77). The inflammatory response following TBIs starts within minutes of the injury (78). Resident brain microglia are the first to activate and migrate toward the focal injury (79, 80). Within hours of injury, neutrophils arrive at the injury site to begin clearance, followed by macrophages 1–2 days later (81). After a TBI, the levels of various cytokines undergo a pronounced increase, which typically peaks hours or days after the injury (82, 83).

Blood-brain barrier (BBB) disruption frequently occurs after TBI and can last from days to years after head trauma (84, 85). Increased BBB permeability is considered a key mechanism in TBI secondary injury, and is involved in prolonged inflammatory responses, delayed neuronal dysfunction, and cell death (85). Additionally, damage to cell lining of the BBB leak compounds usually confined within the brain into the periphery, exposing innate, and adaptive immune cells to neurological antigens. Some researchers have suggested that acute TBI may trigger brain tissue-targeting autoimmunity (86), and indeed, acute TBI patients have developed autoreactive antibodies and T-cells within the periphery that are capable of detecting and reacting with brain-derived components years after the initial injury (81). Components of the BBB (i.e., astrocytes, pericytes and endothelial cells) are susceptible to the effects of the injury (87), but underlying molecular changes that lead to BBB disruption following TBI are not completely known.

Pathophysiological mechanisms of TBI that result in AD-like neurodegenerative processes remain poorly understood, but neuroinflammation leading to neurodegeneration is a likely candidate. AD pathology is characterized by intra-neuronal neurofibrillary tangles of hyperphosphorylated tau (p-tau) and deposits of extracellular Aβ, which likely relate to the dysfunction of brain clearance mechanisms (88, 89). Aβ deposition is regulated by an equilibrium between Aβ production and clearance. Chronic traumatic encephalopathy (CTE) and CTE-like neurodegeneration involve the progressive buildup of p-tau and neurofibrillary tangles (NFT). Neuroinflammation, with the presence of activated microglia and astrocytes, has also been implicated in AD as well CTE. Similarly, long-lasting increases in microglia and astrocyte reactivity, in addition to elevated levels of proteins associated with neurodegeneration (e.g., tau, Aβ42, and Aβ40), have also been described in TBI (90–92). In TBI, as well as in neurodegenerative diseases, microglia and astrocyte activation is a double-edged sword. Microglia and astrocyte activation can elicit protective mechanisms, but their persistent activation can also trigger deleterious processes and worsen tissue injury (75, 93). At a certain timepoint during disease progression, glial cells assume a useful role, then progress into a dysfunctional cell that ultimately becomes harmful.

Further understanding the neuropathology of TBI and mechanisms underlying long-term consequences of head injuries is fundamental to develop novel and effective clinical interventions. Investigating the emerging role of EVs in TBI and related pathologies may fill important knowledge gaps. However, as discussed next, the term EV encompasses a variety of vesicle types, which are yet to be fully characterized and may play distinct roles in TBI.

## Heterogeneity of Extracellular Vesicles

EVs are heterogeneous in size, content, biogenesis, and membrane composition, which suggests variability in biological function. Terms used to classify EVs include exosomes, ectosomes (microvesicles or microparticles), apoptotic bodies and oncosomes (29, 94). EVs include different populations of vesicles that can be categorized according to their biogenesis mechanisms in exosomes (derived from endocytic membranes) and ectosomes (assembled in the plasma membrane) (95). Exosomes are smaller (30–150 nm) than ectosomes (50–1,000 nm), but size alone does not determine the population which adds to the challenge of distinguishing EV subtypes (29, 31, 96, 97). Exosome precursors are called intraluminal vesicles (ILVs) which are formed via the inward budding of the membrane of endocytic cisternae. The accumulation of ILVs in the endocytic cisternae forms multivesicular bodies (MVBs). When MVBs fuse with the plasma membrane, the released ILVs are called exosomes. EV biogenesis has been reviewed in detail elsewhere (95). Apoptotic bodies are vesicles that are also shed from the plasma membrane during apoptosis (98). The term oncosomes (100–400 nm) is applied to vesicles that carry abnormal macromolecules such as oncogenic proteins (99).

Exosomes have membranes abundant in tetraspanins (e.g., CD9, CD63, CD81) that are important for trapping membrane and luminal proteins and lipids (e.g., cholesterol and sphingomyelin) (94, 95, 100). Exosomal membrane also contains adhesion proteins (e.g., L1 cell adhesion molecule, L1CAM, which is considered a neuron-specific marker), integrins, heat shock proteins (HSPs), tumor susceptibility gene 101 protein (Tsg101), and ALG-2-interacting protein X (Alix), among others (31). The membrane of ectosomes is rich in glycoproteins, metalloproteinases and some receptors (101). Exosomes and ectosomes contain many proteins and nucleic acids (mRNAs, miRNAs and other non-coding RNA) in their lumina (102). Exosome cargo is enriched for miRNA and their membrane offers protection against RNAases that degrade free RNA, providing higher stability for miRNAs in body fluids and during experimental manipulation (102).

Exosome and ectosomes are both found in extracellular fluids, such as blood, and may be produced by the same cell types (103, 104). EVs with a similar size as exosomes can bud at the plasma membrane, and exosomes themselves are a heterogeneous population with variable sizes (100). Thus, determinations of size and density of exosomes should not be used as the only criteria to determine the presence of exosomes in a sample. Because exosome membrane is enriched in tetraspanins, they are frequently used as exosome markers (31, 100). However, tetraspanins might also be present in other subpopulations of EVs (100). Additionally, EVs derived from distinct cell types differ not only in their cargo content but also in membrane proteins, allowing for the use of antibodies against specific protein markers to enrich samples for EVs that originated from specific cells (100).

The existence of cell-derived vesicles has been known for decades. Platelet-derived vesicles were described by Wolf in 1967 (105). He reported that plasma free of platelets contains a material he called platelet-dust, which he isolated by ultracentrifugation and that contained coagulant properties. The term exosomes was first proposed by Trams et al. (106), referring to vesicles “exfoliated” from neoplastic cell lines. Examining these vesicles under the electronic microscope, Trams et al. (106) reported an average diameter ranging from 500 to 1,000 nm and the frequent presence of a second population of vesicles 40 nm in diameter. In 1987, the term exosomes was used to describe vesicles released from the plasma membrane and originated from multivesicular bodies that fused with the plasma membrane of reticulocytes in cell culture (107). Subsequent studies showed the release of exosomes from different cell types and the presence of MHC class-II on the membrane of these vesicles (108–111). Exosomes released from human and murine B lymphocytes induced antigen-specific MHC class-II restricted T cell responses, suggesting a role for exosomes in antigen presentation *in vivo* and in immunological responses (108).

The interest in exosomes, and more recently other EV types, has increased during the last decade, resulting in an extensive and rapidly growing literature, making it challenging to separate evidence-based information from assumptions and hypothesis. A wealth of information regarding exosomes and other EVs can be found in online resources such as ExoCarta (http://www.exocarta.org) (112) and Vesiclepedia (http://www.microvesicles.org). In an effort to establish minimal requirements for the definition of EVs and their functions, the International Society for Extracellular Vesicles (ISEV) has published a set of guidelines (31, 113). Nevertheless, terminology and classification of EVs, including the size range associated with specific EV types, is highly variable in the literature. Further understanding of EV roles in healthy tissues and pathological processes, in addition to technical advancements in the field, may shed light on the functional significance of EV heterogeneity and allow further characterization of distinct vesicle subpopulations. Concentrations and content of specific EV subpopulations could be analyzed in TBI patients, examining relationships between biomarker levels in each EV subpopulation and TBI recovery.

## Extracellular Vesicles in the Central Nervous system and Neurological Diseases

The secretion of EVs used to be understood to be a means of elimination of proteins and unwanted molecules from the cells (114). Currently, EVs are considered promising biomarkers and delivery systems for therapeutics and a new form of cell-to-cell communication with roles in an expanding list of diseases and conditions such as cancer, inflammatory bowel diseases, obesity and diabetes, rheumatoid arthritis, and neurological diseases (115). In TBI, possible roles for EVs are only beginning to be explored. Studies investigating EVs in TBI will be discussed in the next section. Here, we briefly discussed evidence suggesting a role for EVs in the brain and neurogenerative diseases, which provides insight into the possible relevance of EVs in TBI.

EVs are released by all major cells in the CNS, including neurons, astrocytes, microglia and oligodendrocytes (116–118). Roles of EVs in brain physiology and disease are only beginning to be understood. Studies have suggested roles for EVs in elimination of waste (119) and cell-to-cell communication (119–121). A subpopulation of MHC class -II-negative microglia has been shown to internalize EVs secreted by oligodendrocytes *in vitro*, which suggests a role for EVs in the pathogenesis of autoimmune diseases that include the transfer of antigens from oligodendrocytes to immune cells (119). A bidirectional communication between neurons and oligodendrocytes involving EVs has also been reported: the release of the glutamate by neurons regulates the secretion of EVs by oligodendrocytes, which are internalized by neurons (122).

In AD, EVs have been hypothesized to be involved in the lateral and long-distance propagation of tau as well as in a number of mechanisms associated with AD pathogenesis as previously reviewed elsewhere (123, 124). Importantly, proteases that contribute to the biogenesis of Aβ fragments have been found in EVs (125–127). Nevertheless, while EVs are likely associated with the progression of AD, they might also be part of protective mechanisms as they are a part of clearance processes in the brain (128, 129). Indeed, EV surface carries insulin-degrading enzyme, which also degrades Aβ (128). EVs are also believed to be a potential source of biomarkers for AD, as well as other neurodegenerative diseases such Parkinson's disease, CTE and Creutzfeldt-Jacob disease. Proteins such as Aβ, tau, α-synuclein and prions are found in EVs (123, 130, 131). Similarly, elevated levels of molecules such as Aβ and tau in EVs might serve as biomarkers for neurodegenerative changes after TBI. Levels of miRNAs in EVs may also be used to reveal underlying signaling mechanisms and serve as biomarkers. Accordingly, changes in miRNA expression, including EV miRNAs, have been linked to aging and age-related diseases (132), and targeted inhibition of miRNAs may have therapeutic effects (133).

As previously discussed, neuroinflammation characterized by glial activation and cytokine release is an important element of neurodegenerative diseases and TBI pathology in acute and chronic phases. Following TBI, peripheral blood levels of diverse cytokines undergo a pronounced increase, which typically peaks hours or days after injury (82, 83). Higher acute blood levels of interleukin (IL)-6 (134–137), IL-10 (134, 138, 139), TNF-alpha (137, 140, 141), as well as other cytokines (83) after TBI have been linked to poor outcomes. Similarly, in chronic TBI, increased blood levels of IL-6 and TNF-α relate to TBI symptoms in military personnel (137, 142). Interestingly, recent studies suggest that cytokines mediate cell-to-cell signaling not only as a soluble factor, but also via a system mediated by EVs (35, 143). Cytokines associated with EVs (surface-bound and encapsulated) are biologically active (143). Researchers have hypothesized that cytokines found on the EV surface may interact with cell-specific receptors facilitating cell-to cell communication (144). A recent study investigating eight different biological systems (e.g., tonsillar explants, amnion explants, T cells, monocytes) suggested that cytokines are released in a soluble (free) form or associated with EVs depending on the physiological context. Authors suggested that systems involving long-distance communication tend to release more EV-associated cytokines (143). Accordingly, EVs are likely implicated in long-distance communication between brain and peripheral tissues (101).

Studies in TBI and AD found higher inflammatory protein markers in EVs isolated from peripheral blood, suggesting a role of EVs in neuroinflammation (35, 145). EVs secreted from monocytes are also thought to influence neuroinflammation by facilitating the exchange of miRNA and proteins (146). Differential regulation of miRNAs associated with peripheral circulating EVs have been described and will be discussed in the following section. Moreover, EV encapsulated miRNAs can deliver genetic material to recipient cells, impacting their gene expression (147). Determining levels of inflammatory proteins and miRNAs in EVs may be used to reveal underlying signaling pathways and serve as biomarkers of specific disease mechanisms.

**Figure 2:**
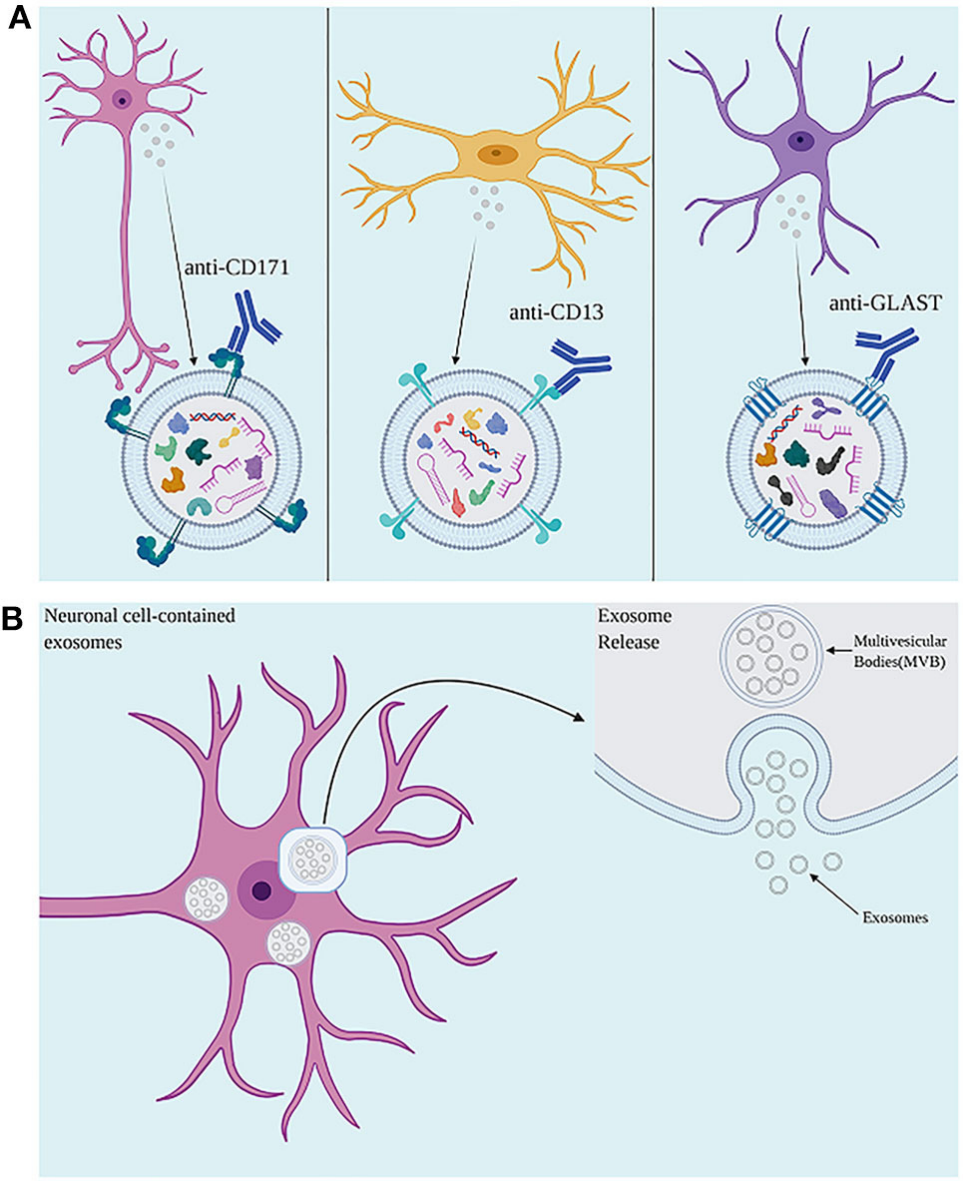
**(A)** Use of antibodies against cell surface proteins present on exosomes allows for isolation of neuronal-, microglial-, and astrocyte-derived exosomes. **(B)** The accumulation of intraluminal vesicles (ILVs), exosome precursors, forms Multivesicular bodies (MVBs). MVBs fuse with the plasma membrane, releasing exosomes to the extracellular environment. Created with Biorender.com.

In TBI and other neuroinflammatory conditions, central inflammation as well as responses from the peripheral immune system are observed (148). As EVs can cross the BBB, EVs originated from the CNS can be isolated from the peripheral circulation (35, 145). Antibodies against proteins located in the EV membrane can be used to isolate EVs of specific cell types from serum, plasma, and other bodily fluids (35, 37, 145), allowing the investigation of mechanisms involving distinct brain cell types in a minimally invasive manner (Figure 2). Several studies have successfully measured inflammatory proteins in neuron-derived (NDE) and astrocyte-derived (ADE) EVs isolated from peripheral blood (35, 37, 149, 150). Neural cell adhesion molecules NCAM and L1CAM (CD171) have been used as targets to select NDE due to their relatively specific expression in neural tissue on derived from cultured neurons (35, 151). To enrich EV samples for ADEs, glutamine aspartate transporter (GLAST) antibody has been used (150). A study in AD patients reported higher levels of classical and alternative complement pathway proteins in ADE when compared to matched controls, suggesting the existence of signaling mechanisms involving inflammatory mediators released by activated astrocytes via EVs (150). This approach could also shed light on the roles played by distinct cell types in the body in response to a TBI as well as distinguish between the central or peripheral origin of proteins and miRNAs found in blood, in TBI and other diseases.

In addition to levels of specific proteins and signaling molecules in EV cargo, studies have evaluated changes in EV concentration in the blood after TBI (35, 149). Decreases in the concentration of NDEs have been reported in the acute but not chronic phase of TBI as measured by particle counts and concentrations of EV markers (149). Accordingly, another study found no significant differences in EV counts when comparing participants with chronic TBI to those with no TBI history (35). Higher EVs counts in acute, but not chronic TBI phases, may reflect mechanisms triggered shortly after injury and that are no longer present at later timepoints. Pathological processes triggered by a TBI vary according to aspects such as time after the injury and its severity (140). Biomarkers that are informative at earlier timepoints and for severe TBIs may not be reliable in chronic or milder injuries due to factors such as lower concentrations. Prospective studies examining longitudinal changes could inform temporal profiles of EV concentration in the peripheral blood. Moreover, as previously discussed, EV populations are very heterogenous. Different EV subpopulations that could likely be characterized by distinct membrane markers and functional roles could be released at different rates depending on factors such as time after TBI, severity of injury, or presence of comorbidities. Study of EV populations in the brain is a new field even though it has witnessed fast technical advancement. EV biogenesis, including protein and miRNA packing, secretion, and their roles in cell signaling are still poorly understood in health and disease. Future studies will likely benefit from technological development in the field to elucidate on the role of EVs in brain pathology. Expanding knowledge on basic mechanisms involved in EV cargo-loading and biogenesis, as well as characterizing distinct EVs subpopulations, are warranted to better understand TBI pathology and unleash their full clinical potential.

## Extracellular Vesicles in TBI

Biomarker studies in TBI have focused on plasma and serum levels of proteins found in brain cells such as tau, p-tau, and neurofilament light chain (NfL) (152, 153); glial fibrillary acidic protein (GFAP), that is released from astrocytes (13); and ubiquitin carboxyl-terminal hydrolase isozyme L1 (UCHL1), a brain-specific deubiquitinating enzyme (154). NfL, GFAP, tau and UCHL1 have been linked to TBI severity, poor recovery, as well as PCS and PTSD symptomology in a variety of populations, including civilians (23, 155, 156), athletes (157, 158) and military personnel (11, 90, 159). Other studies have evaluated levels of inflammatory markers (134–137) as previously discussed in this article. Challenges of measuring blood-based biomarkers include low concentrations in the peripheral circulation. That is the case of tau, which requires highly sensitive platforms to be reliably measured in blood (160). Low levels of brain-derived biomarkers in blood may be attributed to factors such as proteolytic degradation and low permeability of the BBB (160). Furthermore, clearance of interstitial proteins depends in part on the glymphatic system (161), which is dysfunctional after TBI (89), likely contributing to discrepancies between levels of biomarker proteins in the brain and blood.

While circulating concentrations of proteins in blood have important diagnostic potential, it is possible that these same proteins within EVs may be more reflective of biological underpinnings of TBI (162). Specifically, EVs have been linked to important biological functions, such as cell-to-cell signaling pathways associated with inflammatory responses and removal of aggregated and misfolded proteins within the brain (129, 143). Moreover, EVs can cross the BBB and their membrane provides protection to proteins and nucleic acids, likely reducing their degradation in the peripheral circulation (163–166).

Here, we have reviewed studies investigating EVs in TBI. We have also included studies using animal models. Currently available animal models of TBI have limitations, especially for mTBI, which include anatomical differences between the brain of humans and non-human mammals (167–169). These limitations contribute to the challenge of translating new therapeutic approaches from bench to the clinic. Moreover, animal models are limited in their ability to mimic the complex symptomatology of TBI in humans that includes cognitive and affective changes (12, 49, 167). Despite limitations, animal models allow the dissection of injury mechanisms and use of genetic manipulation, providing opportunities to develop novel therapies and evaluate them before human testing. A summary of clinical and pre-clinical studies is provided in Table 1, a selection of studies has been discussed below to illustrate approaches that have been used to investigate EVs in TBI.

**Table 1:** Extracellular vesicles studies in traumatic brain injury.

**Reference**	**Organism**	**Cohort**	**Focus**	Measured exosomal cargo^*^
Kawata et al. (170)	Human	Sports Related Concussion (acute and post-acute)	Plasma and brain, neuron, astrocyte, microglia- derived EVs	NfL, tau, SNAP25, GFAP, MBP
Mondello et al. (171)	Human	Moderate-to-severe TBI (acute through sub-acute)	Serum exosomes	**GFAP**, NfL, **total tau, UCHL1**
Goetzl et al. (149)	Human	Sports Related Concussion (acute and post-acute mTBI)	Plasma, neuron-derived exosomes	**UCHLI, Aβ42, AQP4, and many others**
Winston et al. (37)	Human	Military-related mTBI (post-deployment sampling)	Neuronal- and astrocyte- derived exosomes	**Aβ42**, **NRGN**, NfL, total tau, p-T180-tau, PS396-tau
Kenney et al. (33)	Human	Military-related chronic repetitive mTBI	Plasma exosomes	**p-tau, total tau**
Gill et al. (35)	Human	Military-related chronic mTBI	Plasma neuron-derived exosomes	**tau**, **Aβ42**, TNF-alpha, IL-6, **IL-10**
Stern et al. (172)	Human	Sports Related Concussion (acute and post-acute)	Plasma exosomes	**tau**
Muraoka et al. (173)	Human	Sports-related TBI (post-acute)	CSF, EVs	**p-tau, total tau**
Goetzl et al. (174)	Human	Acute TBI	Plasma and serum neuron-derived exosomes	**SYNPO**, NSE, mitochondrial cytochrome c oxidase
Wang et al. (175)	Human		Exosomes	**p-tau, total tau**
Ghai et al. (34)	Human	Blast related chronic military TBI	Plasma EV's	32 miRNAs in plasma; **45 miRNAs in EVs**, concentrations of C-reactive protein (CRP) and membrane metalloendopeptidase (MME) elevated in chronic mTBI samples
Ko et al. (176)	Human, mouse		Brain-derived EVs	
Ko et al. (177)	Human, mouse		Brain-derived EVs	
Wang et al. (34)	Rat	mTBI	Plasma exosomes	**50 miRNAs differentially expressed: 30 up** (miR-9a-3p, miR-29b-3p, miR-106b-5p, miR-124-3p, miR-142-3p, miR-181c-3p, miR-195-3p, miR-328a-5p, miR-361-3p, miR-374-5p, miR-434-3p, miR-532-5p, and others), **19 down** (miR-145-3p, miR-221-5p, miR-28-3p, miR-96-5p, miR-9a-5p, and others)^+^
Hazelton et al. (178)	Mouse	Acute TBI	EVs	Selective targeting of macrophage/monocyte populations
de Rivero Vaccari et al. (179)	Rat		Neuron-derived exosomes	
Ge et al. (180)	Mouse	rmTBI	Microglial exosomes	miR-124-3p
Huang et al. (36)	Mouse	rTBI	Microglial exosomes	miR-124-3p
Di et al. (181)	Mouse	rTBI	Microglial exosomes	miR-124-3p
Yang et al. (182)	Rat		Inflammation/neuroprotection/therapeutic value (exosomal miR-124)	miR-124-3p
Harrison et al. (183)	Mouse		EVs	**miR-21, miR-212, miR-146, miR-7a, and miR-7b**
Kim et al. (184)	Mouse		MSC-derived exosomes	
Zhang et al. (185)	Rat		Plasticity/neuroprotection/therapeutic value (MSC-derived exosomes)	
Ni et al. (186)	Mouse		Inflammation/neuroprotection/therapeutic value (BMSC-derived exosomes)	
Sun et al. (187)	Rat		Therapeutic value (NSC-derived EVs)	
Wang et al. (188)	Mouse		Astrocyte-derived exosomes	
Zhang et al. (189)	Rat		Therapeutic value (cell-free exosomes generated by human BMSCs cultured under conventional or 3D conditions)	

Summarized work comprises biomarker, mechanistic as well as therapy-focused publications in clinical studies (top) and animal models of TBI (bottom). TBI, Traumatic brain injury; rTBI, repetitive TBI; EVs; CSF; MSCs, mesenchymal stem cells; BMSCs, Bone-marrow derived mesenchymal stem; NCS, neural stem cells; 3D, 3-dimensional; miR, microRNA; NfL, neurofilament light chain; UCHL1, Ubiquitin C-Terminal Hydrolase L1; Aβ42; AQP4, Aquaporin-4; NRGN, Neurogranin; p-tau, phosphorylated tau; TNF-alpha, Tumor necrosis factor-alpha; IL-6, Interleukin 6; IL-10, Interleukin 10; SYNPO, Synaptopodin; NSE, neuron-specific enolase. + Underlined miRNAs were noted as more relevant to study.

* *Molecules measured in exosome cargo are described when applicable (Statistically significant analyses marked in bold)*.

### Serum and Plasma Extracellular Vesicles

Studies have examined levels of biomarkers in EVs isolated from either serum or plasma, without enriching samples for specific EV subtypes. Mondello et al. (171) explored longitudinal trajectories of serum EV levels of proteins and their free-circulating counterpart in moderate-to-severe TBI for up to 5 days after injury. Authors found differences in dynamics of free-circulating and EV proteins. Total tau (t-tau) and UCH-L1 levels in EVs were substantially increased immediately after injury and quickly dropped. For EV UCHL1, two distinct groups were identified, with early increase in UCHL1 levels in both. In one of the groups, a decline of EV UCHL1 levels was observed in the first 2 days. The second group had substantially higher early concentrations of EV UCHL1 and a subsequent decrease, which was followed by a secondary peak with very high concentrations. This trajectory strongly predicted early mortality (within 3 days). Higher levels of EV NfL and GFAP were observed in those with diffuse injury when compared to those with focal lesions. Correlations between EV and free-circulating levels of t-tau and UCHL1 were initially weak, and worsened at later time-points. Alternatively, correlations for NfL and GFAP were strong and improved overtime. These findings highlight the complexity of the relationship between free and EV levels of proteins, and the need for studies comparing both.

In sports-related mTBI, Stern et al. (172) found that tau in plasma EVs was elevated in former National Football League (NFL) players who sustained mild repetitive TBIs (rTBIs) when compared to controls, suggesting its potential use as a predictive biomarker of CTE. Similarly, Kenney et al. (33) analyzed plasma EV levels of t-tau and p-tau in Veterans with a history of military-related mTBI. Higher EV levels of t-tau and p-tau were found in Veterans with rTBI compared to Veterans with two or less mTBIs, or no mTBI. Kenney et al. (33) also found that higher levels of EV t-tau and p-tau were correlated with more severe PCS and PTSD symptoms, whereas Stern et al. (172) observed that the number of tau-positive plasma EVs correlated with worse cognitive function, but not measures of mood and behavior. Kenney et al. (33) also compared cases of mTBI with loss of consciousness (LOC)/PTA; mTBI with alteration of consciousness (AOC) only, without LOC or PTA; and controls without history TBI, but found no significant differences in concentrations of t-tau or p-tau. These findings suggest that elevations in EV t-tau and p-tau are linked to history of multiple lifetime mTBIs, rather than presence of LOC/PTA after the injury. Future studies should include multiple timepoints, evaluations of cognitive function, mood, and neurobehavioral symptoms. Additional studies are also warranted to confirm the potential of t-tau and p-tau to predict severity of TBI symptoms in individuals with chronic rTBI, and risk for CTE and other tauopathies (6, 71).

Tau is a microtube-associated protein, with multiple isoforms generated by alternative splicing (190). Tau phosphorylation regulates tau function, but hyperphosphorylated tau forms aggregates and intraneuronal neurofibrillary tangles that result in neurodegenerative changes (129). Mechanisms underlying neurodegenerative changes in TBI are poorly known, but they may share elements with tauopathies. In neurodegenerative diseases, increased extracellular levels of tau could be attributed to passive release of tau from dead or dying neurons (191). However, EV-mediated secretion of tau in tauopathies has been shown (129, 191). In mild AD, EV-associated relative to free tau is elevated in CSF (129, 191). Challenges in the study of tau as a biomarkers include the low levels of tau in peripheral circulation, which requires high-sensitivity platforms to obtain reliable measurements (150, 157). Furthermore, tau is also expressed in peripheral tissues such as muscle, liver and kidney (192). Sample enrichment for NDEs could improve tau measurements in peripheral blood and allow the analysis of levels of tau derived from CNS, rather than other tissues. Studies that measured levels of biomarkers in NDEs and ADEs are discussed next.

### Plasma Neuron-Derived and Astrocyte-Derived Extracellular Vesicles

Gill et al. (35) evaluated the levels of tau, Aβ40, Aβ42, IL-6, IL-10, and TNF-alpha in plasma NDEs. EVs were enriched for neuronal injury by using an immunoprecipitation method with L1CAM (CD171) antibody. Elevated levels of NDE tau, Aβ42, and IL-10 were found in Veterans with chronic mTBI compared to controls, with elevations in tau being the most related to PCS symptoms endorsed within the mTBI group (35). Despite relatively small sample size, Gill et al. (35) showed that protein markers of neurodegeneration can be measured in NDEs isolated from the blood of chronic mTBI patients, which is associated with the severity of symptoms, suggesting the potential of NDEs as prognostic biomarkers in chronic mTBI. Prospective studies are needed to further examine longitudinal changes in NDE, and their potential as prognostic markers for PCS.

Elevated levels of plasma NDE Aβ42 have also been detected in service members with mTBI exposure at less remote timepoints in a study by Winston et al. (37). In this study, plasma NDE as well as ADE proteins were measured in service members within 3–6 months of deployment (37). EVs were precipitated and enriched for NDE and ADE by using L1CAM and GLAST antibody, respectively, using magnetic beads to immunocapture the proteins that were selected by fluorescent activated cell sorting (FACS). Plasma NDE and ADE levels of Aβ42, Aβ40, neurogranin (NRGN), NfL, t-tau, p-T180-tau, and PS396-tau were compared in service members with deployment-related mTBI to controls with no mTBI history. Higher levels of Aβ42 in plasma NDE and ADE, and lower levels of NRGN in NDE and ADE were found in service members with mTBI exposure; however, no differences in Aβ40, t-tau, NfL, p-T180-tau, and PS396-tau were observed. NDE and ADE levels of Aβ42 and NRGN distinguished service members with mTBI from those with no TBI with moderate sensitive and accuracy (37). Winston et al. (37) also observed that plasma NDE cargo proteins from mTBI samples, but not ADE cargo proteins, were toxic to neuron-like recipient cells *in vitro*.

Goetzl et al. (149) found that levels of proteins in the cargo of plasma NDEs distinguish between acute and chronic sports-related mTBI. Immunoprecipitation in association with L1CAM antibody was also used to enrich samples for neuronal origin. Plasma NDE were collected from athletes within 1 week of sports related TBI, at 3 months or longer following the last of 2–4 mTBIs (chronic mTBI), and in athletes with no prior history of TBI. Plasma NDE proteins assessed between the 3 groups included neurofunctional proteins (Rab-10; annexin VII; UCHL1; AII-spectrin fragments; claudin-5; sodium-potassium-chloride cotransporter-1; Aquaporin-4, AQP4; Synaptogyrin 3, SYNGR3), and neuropathological proteins (Aβ42; P-T181-tau; P-S396-tau; IL-6; prion cellular protein, PRPc). NDE levels of the functional brain proteins were significantly altered relative to controls in acute but not chronic mTBI. In acute and chronic mTBI, elevated NDE levels of neuropathological proteins were observed. The same set of proteins was subsequently assessed by Goetzl et al. (193) in a study of military-related chronic TBI. Plasma NDE protein levels were compared among Veterans assigned into groups based on TBI history and current cognitive impairment (CI). Plasma NDE levels of PRPc, SYNGR3, P-T181-tau, P-S396-tau, Aβ42, and IL-6 were significantly elevated in Veterans with TBI and CI compared with controls with TBI but no CI. Among Veterans without TBI, subjects with CI had significantly elevated levels of PRPc, SYNGR3, P-T181-tau, and Aβ42, in comparison to controls without CI. Taken together, these findings suggest that neuronal Aβ peptides and P-tau species remain elevated for decades after TBI, may be associated with TBI-related cognitive alterations and neurodegenerative changes, and should be considered as potential therapeutic targets.

### Extracellular Vesicle miRNAs

MiRNAs are small, about 21 nucleotides long, non-coding RNAs that function as gene regulators at the post-transcriptional level in eukaryotic cells (194, 195). Pre-miRNA are hairpin-loop precursors that are 60–90 nucleotides long and cleaved into miRNA duplex by the ribonuclease III in the cytoplasm. The mature miRNA negatively regulates gene expression by targeting messenger RNA (mRNA) (196). In TBI, miRNAs have attracted interest as possible biomarkers, and as therapeutic targets.

MiRNAs have been linked to inflammation in several human diseases (197). In a recent study, (34) isolated miRNA from plasma and plasma-derived EVs from Veterans with blast-related mTBI, which were analyzed by using next generation sequencing (NGS). Analysis revealed that 45 and 32 miRNAs were differentially regulated in EVs and plasma, respectively. Pathways functionally associated with differentially regulated miRNAs involved neuroinflammation, BBB integrity, vascular modeling, and neuronal function. Future studies should investigate miRNA changes in response to mTBI caused by other mechanisms, such as blunt head trauma, and at different timepoints after injury.

In a 2018 study, Ko et al. (176) identified a miRNA based panel biomarker to diagnose TBI, both in a mouse model and human TBI. MiRNAs associated with EVs positive for GluR2 (an AMPA receptor subunit) were isolated from plasma of mice exposed to blast overpressure injury. MiRNA profiling in combination with machine learning were used to generate a biomarker panel of seven miRNA (miR-129-5p, 212-5p, miR-9-5p, miR-152-5p miR-21 miR-374b-5p, miR-664-3p) capable of distinguishing TBI patients from healthy controls with high accuracy (176). In a subsequent study, miRNA profiling of GluR2+ EVs across various injury types, severity, and times, allowed Ko to identify distinct TBI signatures across different injury models and post-injury time points and biomarker panels capable of classifying specific states of injury (177). A panel of eight miRNAs were identified for injured mice vs. sham mice. Four were differentially regulated in TBI patients when compared to healthy controls, (miR-203b-5p, miR-203a-3p, miR-206, miR-185-5p) (177).

In a 2016 study, Harrison et al. (183) examined the miRNA cargo of brain-derived EVs isolated from brain injured mice and controls. Decreased expression of miR-212, and increased expression of miR-21, miR-146, miR-7a, and miR-7b were observed in injured mice at 7 days after controlled cortical impact (CCI) relative to controls, with miR-21 showing the largest change between the groups (183). Notably, the authors found that the expression of miR-21 was largely localized to neurons near the lesion site and, notably, that adjacent to these miR-21-expressing neurons were activated microglia (183). This study reveals potential mechanisms of cell-to-cell communication as the increase in miR-21 in EVs with the elevation of miR-21 in neurons, suggests that miR-21 is secreted from neurons as EV cargo.

### Microglial Extracellular Vesicles

History of rTBI is believed to make the brain more susceptible to pathological processes as a consequence of a head trauma, which might be at least partially mediated by microglial cells (70, 198). After a brain injury, microglia are hypothesized to remain in a heightened inflammatory status or primed. The primed microglia have a lower threshold for response to events that disrupt the brain physiology (199). Moreover, recurrent head trauma has been linked to the postmortem diagnosis of CTE in contact-sports athletes and in the military. Neuroinflammation is observed in CTE brains, with large increases in the number of activated microglia in the white matter (200). Microglia-derived EVs (MDEs) have been linked to AD. As previously discussed, microglia activation may have beneficial effects in earlier stages after injury, but later become detrimental. However, the role of miRNAs in microglial EV on regulation of TBI-neurodegeneration is still unclear.

In a mouse model of rTBI, analysis of MDE miRNAs revealed that miR-124-3p played a protective role in TBI-related recovery processes by promoting M2 polarization in microglia and repressing neuroinflammation (36). In support of these findings, Yang et al. (182) showed that EV miR-124 treatment enhanced hippocampal neurogenesis and functional recovery by promoting the M2 polarization of microglia, the effect of which was produced through inhibition of the Toll-like receptor 4 (TLR4 pathway). In a subsequent study, Li et al. (181) showed that increased miR-124-3p in MDE promoted neurite outgrowth via miR-124-3p transfer into neurons, thereby inhibiting neuronal autophagy and protecting again against nerve injury.

In a 2020 study of rTBI, miR-124-3p levels in MDE were found to be significantly altered in the acute, sub-acute, and chronic phases following the injury (180). Intravenous administration of MDE with upregulated miR-124-3p alleviated neurodegeneration in repetitive scratch-injured neurons, the effects of which were exerted by miR-124-3p targeting RelA, an inhibitory transcription factor of apolipoprotein E (ApoE) that promotes β-amyloid proteolytic breakdown, thereby inhibiting β-amyloid abnormalities (180).

Studies analyzing the content of MDEs cargo have performed the enrichment from cultured microglia (180), instead of peripheral blood samples. To our knowledge, no study have examined MDEs in clinical samples, likely for a lack of antibodies shown to distinguish EVs derived from microglia, from those derived from peripheral macrophages. Cell surface markers used to identify myeloid cells in the CNS are expressed by microglia as well as macrophages (201). Evidence suggests a role of MDEs in neurodegeration and neuroinflammation, making microglial EVs a likely candidate biomarker in TBI.

## Methodological Challenges

In this section we will discuss some of technical challenges such as biomarker source, isolation methods, and diversity of EV populations encountered in EV-based biomarker research using clinical samples in TBI.

### Source

Blood is a source for biomarkers, and is frequently used in clinical diagnostics (202, 203). Exosomes have been shown to maintain the majority of their protein and nucleic contents in serum and plasma, with fresh plasma considered the best source of intact exosomes (204). Muller et al. (204) concluded that both plasma and serum are equally comparable sources of EVs when evaluating total protein recovery and morphology of isolated EVs. However, when looking at fresh vs. frozen plasma, fresh plasma yielded less protein aggregation (more purity) and morphologically intact EVs (35). Although a single freeze-thaw cycle with a storage of 1 year did not affect size and concentration of EVs in the study by Yuana et al. the authors noted changes to the membrane phospholipid distribution suggesting increase in coagulation (205), whereas Muller et al. (204) found increase in protein aggregation after thaw/freeze cycles. Additional studies have also shown that exosomes stored at −80 or −20°C in plasma are more stable yielding higher recovery compared to storage at 4°C after 90 days of storage (206, 207).

Yet plasma remains the most heterogenous of all body fluids, being abundant in platelets, albumin, lipoproteins, fibrinogens and many other proteins, also making it the most challenging source for exosomal purity. Complicating matters more, much of blood plasma and serum repository samples follow different collection and handling procedures. For example, because many EVs are platelet derived, it is ideal to have platelet poor plasma (PPP) or platelet free plasma (PFP) so that samples can be used in other cell-focused exosome research. Pre-handling of the blood that includes the process of venipuncture, time between blood draw and initial centrifugation, and subsequent centrifugation speeds are all important factors that will affect EV recovery and purity. Lacroix et al. (208) investigated these factors and concluded that using larger needle size, discarding initial few milliliters of blood, decreasing time delay for initial blood processing under 2 h, introducing two subsequent centrifugations of 2,500 g for 15 min yielded better recovery of EVs from plasma (208).

Bypassing these steps enriches the collected plasma with platelets, making non-platelet EVs isolation increasingly difficult. One common technique pretreats plasma with thrombin to remove platelets, however, a recent study investigated the differences between centrifugation vs. thrombin methods and found substantial loss of vesicles in the fibrin clot when treated by the thrombin (209). Specifically, the authors demonstrated the fibrin clot to become activated by thrombin treatment, leading to entrapment of EVs in the clot and a reduction of total sample EVs. As previously described, repeated low speed centrifugation allows higher recovery of vesicles (208). Briefly, the initial vial of blood should be discarded to avoid release of platelets activated by venipuncture, and the collection tubes are then centrifuged at 3,000 g × 15 min to obtain PPP. When looking at neuronal biomarkers, researchers must be cautious in interpreting results when improper collection or processing measures were used.

### Isolation Method

As the field of extracellular vesicle research exponentially grows, experts often debate the “best” isolation technique (97). While each method unequivocally offers certain advantages, the selection of one over another is driven by the aims of the project. The choice of isolation technique is dictated by a variety of factors, including sensitivity, specificity, cost, personnel, sample, and time constraints. For example, a researcher looking to characterize exosome morphology may select multi-step ultracentrifugation, whereas a researcher conducting clinical trials would be more inclined toward a large throughput method, such as polymer-based precipitation or high-throughput size exclusion chromatography (SEC) (210, 211). When analyzing hundreds of patient samples, methods such as ultracentrifugation of microfluidics can be cumbersome. Current TBI research has used a combination of polymer-based precipitation and immunoaffinity methods to pull down neuronal exosomes. A detailed table comparing different separation or isolation methods described in position statement by MISEV suggests that high recovery and high specificity may not yet be achievable (100). While polymer solutions allow for isolation of EVs from relatively small sample volumes with high recovery, it has low specificity (100) leading to co-precipitation of co-isolated contaminants such as non EV proteins and polymer requiring post-isolation clean-up or purification methods. A survey among EV researchers showed that most common additional purification steps post EV isolation included ultracentrifugation and density gradient centrifugation (212). Without additional cleaning steps, it is difficult to characterize the morphology and composition of the derived.

Due to the heterogeneity of sample preparation and challenges related to co-isolated contaminates, the MISEV has a suggested set of minimal reporting guidelines. For example, to characterize EVs they recommend demonstrating presence of: (1) non-tissues specific tetraspanins (e.g., CD81, CD63), (2) membrane proteins (e.g., TSG101, ALIX), and minimal presence or absence of (3) source specific contaminants (albumin, APOA1/2). (100). Several studies in TBI demonstrated presence of EV markers using western blot (e.g., CD63, CD9, HSP70) (213, 214) and Chen et al. (214) utilized western blotting (WB) to show presence of GJA1-20K from astrocyte-derived EVs which facilitated neuronal recovery. MISEV also recommends characterization of EV morphology using transmission electron microscopy and characterization of EV size and concentration, most often conducted using nanoparticle tracking analysis (100). The main aim is identification of biomarkers with good specificity, sensitivity, and reproducibility. The latter may pose the biggest challenge due to variations in the sample processing, incubation times, plasma pre-cleaning steps, and variability in protocols across different laboratories.

Perhaps one of the most exciting directions for identifying TBI biomarkers is diversity within EV populations. Recent publications (215, 216) discuss limitations of previously established notions of an “exosome” and the importance of distinguishing EV subtypes. Different isolation methods can eliminate a subset of exosomes, whether smaller or larger, that contains important diagnostic information. Experts debate whether subtype classification should be done through biogenesis pathways or EV size (144), however, isolation of specific exosomal categories is still being developed and classification is actively being determined.

## Discussion

TBI is a heterogenous injury with highly variable clinical presentation and recovery patterns. TBI can lead to lasting or late-in-life neurobehavioral sequelae, cognitive and affective symptoms, and is associated with increased risk of developing neurodegenerative diseases. Reliable biomarkers for TBI could improve diagnosis and therapeutic monitoring of individuals who have sustained head injuries. Determining those who are most at risk for neurodegenerative processes and chronic symptoms after a TBI is essential, and identifying underlying mechanisms may provide necessary insights for developing clinical interventions prior to the onset of non-reversible pathological changes.

EVs have been successfully isolated from human serum and plasma from TBI patients, allowing the quantification of proteins and RNAs in their cargo. Blood-based EV biomarkers confer advantages when compared to free proteins and miRNAs, as EVs cross the BBB and their membrane protects the cargo from degradation. Moreover, antibodies against proteins located in the EV membrane can be used to isolate EVs of specific cell types in the peripheral circulation. This approach can be applied to improve measurements for proteins such as tau, which is found at low concentrations in the peripheral circulation, and can be released by the brain as well as peripheral tissues. It also provides a powerful tool to distinguish peripheral and central pathological processes, shedding light on mechanisms associated with neuroinflammation and peripheral immune responses in TBI. Finally, identifying proteins and miRNAs originated from distinct cell types of the brain could improve our understanding of how specific cell types respond to the injury, and underlying signaling mechanisms. Moreover, abundant evidence suggests a role of EVs in the physiological and pathological processes in the CNS, including cell-to-cell signaling between distinct cell types in the brain and clearance processes, eliminating unwanted biological material.

In neurodegenerative diseases, EVs are thought to contribute to the spread of pathogenic proteins, including lateral and long-distance propagation of tau. Mechanisms involving EVs in neurogenerative diseases may provide insight into the possible relevance of EVs in TBI pathology, which is still poorly understood. Neurodegeneration and neuroinflammation are major elements in the neuropathology of TBI as well as neurological diseases. Biomarkers of AD such as Aβ42, t-tau, p-T180-tau, and PS396-tau among others have also been found in EVs isolated from TBI patients at higher levels than controls. Studies have shown higher levels of EV tau and Aβ42 in populations with history of multiple mTBIs, which were linked to the severity of neurobehavioral symptoms. NfL, which is considered a marker of neuronal injury and degeneration, is elevated in many neurodegenerative diseases. Moreover, higher levels of EV nfL and GFAP, an astrocyte marker, were associated with diffused injury when compared to focal lesions in patients with moderate-to-severe TBI.

Monitoring brain injury and associated symptoms using blood-based biomarkers is a safe and relatively inexpensive method. A fast-growing literature suggests the potential of EVs isolated from peripheral blood as TBI biomarkers. Nevertheless, the study of EVs in health and disease is still in its infancy; there are technical limitations and a lack of standards regarding terminology and vesicle characterization. Future studies may benefit from technological development in the field to shed light on the role of EVs in brain pathology.

## Author Contributions

VG and JG contributed to the conception of the review, coordinated writing efforts, and edited the final article version. VG wrote the Introduction. JL and VG wrote the TBI-related Symptoms and Associated Disorders. VG and JA wrote the Neuropathology of TBI. VG wrote Heterogeneity of EVs, EVs in the Central Nervous System and Neurological Diseases, and the Discussion. CD and VG wrote the Extracellular Vesicles in TBI. DS wrote the Methodological Challenges. CD, JL, SM, and VG constructed the table. JA produced the figures. All authors contributed to critical revision of the manuscript, read and approved the submitted version, and agree to be accountable for all aspects of the work in ensuring that questions related to the accuracy or integrity of any part of the work are appropriately investigated and resolved.

## Conflict of Interest

The authors declare that the research was conducted in the absence of any commercial or financial relationships that could be construed as a potential conflict of interest.
